# Feature Extraction Method for Blasting Vibration Signal Based on WOA-VMD and Hilbert Spectrum Analysis

**DOI:** 10.3390/s26216780

**Published:** 2026-10-26

**Authors:** Long Zhang, Zengyue Li, Shiqian Song, Xiaofei Huang, Xuejie Jiao, Zequn Ba

**Affiliations:** Northwest Institute of Nuclear Technology, Xi’an 710024, China; zhanglonglxy@163.com (L.Z.);

**Keywords:** blasting vibration signal, Hilbert spectrum analysis, signal feature extraction, VMD, wavelet threshold de-noising, WOA

## Abstract

Extracting the instantaneous time–frequency distribution and energy evolution of a blasting vibration signal is a critical approach for investigating rock mass structural stability and blast-induced seismic effects. This paper proposes a blasting vibration signal feature extraction method based on whale optimization algorithm–variational mode decomposition (WOA-VMD) and Hilbert spectrum analysis. First, WOA optimizes VMD parameters (decomposition layer K, penalty factor α) to yield the optimal parameter set, and the optimized VMD then decomposes vibration signals into intrinsic mode functions (IMFs). Second, a quantitative multiscale permutation entropy (MPE)-based criterion is introduced to discard noise-dominated invalid components, eliminating subjective judgment errors, while wavelet threshold denoising is applied to valid components for signal reconstruction. Finally, Hilbert spectrum analysis is performed on the reconstructed signals, so that denoising serves as an intermediate check and the outcome is the distance dependence of the time–frequency features and energy evolution. The method is applied to measured vibration signals from three single-hole coal mine blasting experiments. Results indicate that optimized VMD exhibits stronger adaptability in frequency-domain segmentation, significantly mitigating mode mixing. The MPE-based criterion, combined with wavelet denoising, accurately eliminates noise components, showing robust performance in energy retention, noise suppression, and signal smoothness. Within 700–1200 m from the blasting center, signal energy is predominantly concentrated in the 0.3–0.5 s interval and 0–60 Hz frequency band. As distance increases, both the energy peak and center frequency decay exponentially, while the peak arrival time increases linearly.

## 1. Introduction

In blasting experiments, blasting energy not only works on the surrounding rock mass, but also propagates outward in the form of stress waves. Due to the effects of absorption and scattering by rock mass medium, the energy of the stress wave gradually decays during the propagation process, which manifests macroscopically as a decrease in vibration amplitude and a loss of the high-frequency component [1,2]. By utilizing appropriate non-stationary signal analysis methods, the time–frequency distribution characteristics, energy evolution patterns, and other elements of blasting vibration signals can be extracted and analyzed. This is an important approach for studying the stability of rock mass structure and the seismic effects of blasting [3,4,5].

Currently, feature extraction methods for non-stationary vibration signals mainly include fast Fourier transform (FFT), wavelet transform, and Hilbert–Huang transform (HHT). Among these, FFT, as a global time–frequency analysis method, can efficiently obtain the overall frequency distribution of vibration signals. However, it is limited by low time resolution and thus fails to extract local time–frequency characteristics of signals [6]. Short-time Fourier transform (STFT) divides a long-time signal into multiple short-time segments by introducing a sliding window function, thereby partially compensating for the time-resolution limitations of FFT. Nevertheless, the accuracy of its time–frequency analysis is constrained by factors such as the type of window function and window length. When dealing with multiscale complex transient signals, STFT cannot achieve high resolution in both the time and frequency domains [7].

Wavelet transform features time–frequency localization and multiresolution analysis capabilities, and is widely adopted for extracting local time–frequency characteristics of non-stationary signals. However, its limitation lies in the lack of adaptability in selecting wavelet basis function, decomposition level, and thresholding rule. These parameters are often determined based on simulation experiments and prior experience, leading to a certain degree of subjectivity in analysis results [8,9]. In contrast, wavelet packet analysis offers a greater variety of wavelet basis functions, which can effectively enhance the accuracy of signal decomposition and high frequency resolution. Nevertheless, this also increases computational complexity and complicates the interpretation of the results [10,11].

As another important tool for extracting time–frequency features of non-stationary signals, HHT can adaptively decompose signals into several intrinsic mode functions (IMFs) based on local characteristic time scales. By performing Hilbert spectral analysis on the IMFs, the instantaneous correspondence of time, frequency, and energy with clear physical significance can be obtained [12]. As the core component of the HHT framework, empirical mode decomposition (EMD) enables adaptive signal decomposition, but it also suffers from non-negligible drawbacks, including the endpoint effect and mode mixing [13]. Building on EMD, researchers have proposed improved methods such as ensemble empirical mode decomposition (EEMD) and complete ensemble empirical mode decomposition with adaptive noise (CEEMDAN). By adding Gaussian white noise to the signal, these methods enhance the continuity of the signal across multiple scales, mitigating the endpoint effect and mode mixing problems of EMD to a certain extent. However, they also introduce new problems, such as residual auxiliary noise, which increases the signal reconstruction error. Moreover, the algorithm mechanism of repeated decomposition and ensemble averaging significantly increases the computational burden, thereby limiting the efficiency of signal decomposition [14,15].

Alternatively, variational mode decomposition (VMD) reformulates signal decomposition as a constrained variational problem. By iteratively solving for the optimal solution of the variational model, it determines the center frequency and bandwidth of each IMF component, thereby adaptively performing frequency-domain segmentation of the signal and clearly separating the IMFs, which effectively addresses the modal mixing problem inherent in EMD [16]. Further studies have shown that the decomposition performance of VMD is highly dependent on the selection of decomposition layer *K* and penalty factor *α*. The value of *K* determines the granularity of signal decomposition. A smaller *K* value will lead to under-decomposition of the signal, causing multiple frequency components to alias into the same IMF, resulting in mode mixing. Conversely, an excessively large *K* causes over-decomposition, which increases the risk of introducing redundant noise. The penalty factor *α* is used to control the smoothness of the IMFs and impose precise constraints on their bandwidth, ensuring that the decomposed IMFs exhibit compact narrowband characteristics in the frequency domain [17,18,19].

In blasting experiments, the vibration signal typically exhibits complex time-varying characteristics and randomness, making it difficult to achieve ideal signal decomposition performance with empirically preset parameter combinations. For this reason, various swarm intelligence methods, including particle swarm optimization (PSO), genetic algorithm, ant colony algorithm, and whale optimization algorithm (WOA), have been applied to optimize the parameters of VMD [20,21,22,23,24]. However, the aforementioned algorithms exhibit distinct inherent limitations in dealing with optimization problems. For instance, PSO is prone to converging prematurely and suffers from weak global search capability. Genetic algorithms involve multiple parameters, such as crossover rate and mutation rate, where suboptimal parameter configurations can significantly impede the convergence speed. The ant colony algorithm also lacks clear parameter setting rules, and irrational parameter configurations may cause the search process to stagnate or fall into local optimal solutions. Moreover, the execution time and memory requirements of the algorithm increase significantly with the growth of population size and iteration count. 

In contrast, WOA uniformly regulates the search process via a dynamically decreasing convergence factor, achieving an adaptive balance between global search and local exploration. Its unique spiral position update and random walk mechanisms endow the algorithm with a powerful ability to escape local optima. Additionally, the algorithm structure only includes two core parameters, population size and maximum iteration count, which significantly reduces the difficulty of parameter setting during model construction. When facing high-dimensional parameter spaces or nonlinear optimization problems, WOA still maintains satisfactory optimization accuracy and convergence speed, demonstrating stronger generalization ability and high robustness [23,24,25]. Quantitative comparisons support these assertions: WOA outperforms PSO, DE, GSA and FEP on 29 benchmark problems [26], and is more accurate and faster than the sparrow search algorithm, PSO and GA in searching the decomposition layer *K* and the penalty factor α for VMD [27]. WOA-optimized VMD has accordingly been applied to tunnel-portal blasting vibration [25] and to microseismic records [28], where the reported SNR and RMSE were superior to those of conventional decomposition-based denoising.

Nevertheless, the aforementioned studies have predominantly focused on isolated improvements or simple integration of individual stages, such as VMD parameter optimization, signal denoising, or feature extraction, lacking explicit transfer of parameters and criteria between stages. Although some recent studies have begun to couple multiple stages for microseismic or other signal types, the WOA-VMD denoising of tunnel-portal blasting vibration [25] and the multi-stage microseismic denoising scheme [28] still take the denoising index as their end point, and a framework specifically tailored for blasting vibration signal feature extraction has yet to be established. In contrast, the proposed method differs from these schemes not in combining four techniques but in how the parameters and criteria are coupled along the chain. The *K* and *α* optimized by WOA define the mode set on which the MPE criterion then acts, so that selection operates on the same decomposition rather than on an independently parameterized one. Every decision rule is fixed before the measured signals are processed and none is re-tuned against the denoising results, so that the measured case tests the chain rather than calibrating it, and denoising is an intermediate rather than a final result, the reconstructed signal rather than the raw record being subjected to Hilbert spectral analysis. These three points constitute the main contribution of this work. For signal decomposition, the WOA algorithm is employed to optimize the decomposition layer *K* and penalty factor *α* of VMD to obtain the optimal parameter combination. The VMD algorithm with optimized parameters can adaptively achieve accurate frequency-band division, avoiding mode mixing or noise redundancy caused by empirically preset parameters, thereby effectively improving the efficiency and accuracy of signal decomposition. At the level of signal noise reduction, a component selection principle based on multiscale permutation entropy (MPE) is introduced to quantitatively screen noise-dominated components, eliminating the random errors introduced by subjective judgment. Meanwhile, a wavelet threshold denoising method based on the weighted average threshold function is constructed, followed by noise reduction processing and signal reconstruction of the dominant components. Satisfactory denoising effects are achieved in terms of energy retention, noise suppression and signal smoothness. For feature extraction, taking the single-hole blasting experiment implemented in a coal mine as an analysis case, Hilbert spectral analysis is performed on the measured vibration signals at different blast center distances. The instantaneous distribution characteristics of time, frequency, and energy, as well as their variation with the blast center distance, are obtained, verifying the effectiveness of the proposed method. 

## 2. Algorithm Principle

### 2.1. Variational Mode Decomposition

VMD is a signal decomposition method based on variational optimization. Its core principle involves determining the center frequency and bandwidth of each IMF by iteratively searching for the optimal solution of the variational model. This method decomposes a complex non-stationary signal into several narrowband IMFs, thereby enabling effective signal denoising and time–frequency analysis [21,22,29,30]. The main steps of VMD are as follows:

1. Definition of the optimization objective. The VMD algorithm decomposes the input signal *f*(*t*) into a series of modes with different center frequencies by iteratively searching for the optimal solution of the variational model. Each mode can be represented as a frequency-modulated (FM) and amplitude-modulated (AM) signal, as in Equation (1).

*u_k_*(*t*) = *A_k_*(*t*)cos[*ϕ_k_*(*t*)] (1)

where *A_k_*(*t*) is the instantaneous amplitude and *ϕ_k_*(*t*) is the instantaneous frequency.

2. Formulation of the variational problem. An optimization problem is constructed via the variational method to minimize the spectral bandwidth of each IMF. The expression of the variational problem is as follows:

(2) $$\left\{\begin{array}{ll} \underset{\left\{\mu_{k}\right\},\left\{\omega_{k}\right\}}{\min}\; & \left\{\sum_{k=1}^{K}{\left\|\partial_{t}\left[\left(\delta (t)+\frac{j}{\mathrm{\pi t}}\right)\mu_{k}(t)\right]e^{-j\omega_{k}t}\right\|}_{2}^{2}\right\}\\ s.t. & \sum_{k=1}^{K}\mu_{k}(t)=f(t) \end{array}\right.$$

where *δ*(*t*) represents the unit impulse function, *K* is the preset number of decomposition modes, *μ_k_* is the IMF generated by the decomposition, and *ω_k_* corresponds to its center frequency.

The quadratic penalty factor *α* and the Lagrangian multiplier *λ* are introduced to transform the above variational problem into an unconstrained optimization problem. The parameter *α* controls the reconstruction fidelity in the presence of Gaussian noise, while *λ* enforces the exactness of the reconstruction constraint. The augmented Lagrangian function is expressed as

(3) $$\begin{array}{c} L\left[\left\{\mu_{k}\right\},\left\{\omega_{k}\right\},\lambda \right]=\alpha \sum_{k=1}^{K}{\left\|\partial_{t}\left[\left(\delta (t)+\frac{j}{\mathrm{\pi t}}\right)\mu_{k}(t)\right]e^{-j\omega_{k}t}\right\|}_{2}^{2}\\ +{\left\|f(t)-\sum_{k=1}^{K}\mu_{k}(t)\right\|}_{2}^{2}+\langle \lambda (t),f(t)-\sum_{k=1}^{K}\mu_{k}(t)\rangle \end{array}$$

3. Solution of the variational problem. The alternating direction method of multipliers (ADMM) is employed to solve Equation (3), and *μ_k_*, *ω_k_*, and *λ* are updated continuously in the frequency domain.

(4) $${\hat{\mu }}_{k}^{n+1}(\omega )=\frac{\hat{f}(\omega )-\sum_{i\neq k}{\hat{\mu }}_{i}(\omega )+\hat{\lambda }(\omega )/2}{1+2\alpha {\left(\omega -\omega_{k}\right)}^{2}}$$

(5) $${\hat{\omega }}_{k}^{n+1}=\frac{\int_{0}^{\infty }\omega {\left|{\hat{\mu }}_{k}(\omega )\right|}^{2}\mathrm{d\omega}}{\int_{0}^{\infty }{\left|{\hat{\mu }}_{k}(\omega )\right|}^{2}\mathrm{d\omega}}$$

(6) $${\hat{\lambda }}^{n+1}(\omega )={\hat{\lambda }}^{n}(\omega )+\tau \left(\hat{f}(\omega )-\sum_{k}{\hat{\mu }}_{k}^{n+1}(\omega )\right)$$

where *n* denotes the iteration index of the solution process and *τ* is the time step.

The ADMM algorithm converges and the iterative update process terminates when the following convergence criterion is satisfied.

(7) $$\sum_{k=1}^{K}\frac{{\left\|{\hat{\mu }}_{k}^{n+1}-{\hat{\mu }}_{k}^{n}\right\|}_{2}^{2}}{{\left\|{\hat{\mu }}_{k}^{n}\right\|}_{2}^{2}}<\varepsilon$$

where *ε* is the convergence precision.

### 2.2. Whale Optimization Algorithm

The whale optimization algorithm (WOA) is a swarm intelligence optimization algorithm that simulates the hunting behavior of humpback whales in nature. By combining biological behavior modeling with dynamic update mechanisms, the algorithm offers advantages such as fast convergence and strong global search capability [23,24,26,31]. According to its underlying principle, the optimization process comprises three stages: encircling prey, spiral bubble-net attacking and search for prey.

1. Encircling prey stage. In this stage, the whales encircle the prey by updating their positions toward the best individual found so far. It is assumed that the current best solution represents the optimal prey location, and all other search agents iteratively move toward it to refine their own positions. The mathematical model is described as follows:

*D*_1_ = |*C* ⋅ *X_best_*(*t*) − *X*(*t*)| (8)

*X*(*t* + 1) = *X_best_*(*t*) − *A* ⋅ *D*_1_ (9)

where *t* denotes the current iteration number, *X*(*t*) is the position vector of the current search agent, *X_best_*(*t*) represents the position vector of the best solution found so far, and *D*_1_ corresponds to the distance between them. The coefficients *A* and *C* govern the movement patterns of the whale individuals, which are defined as follows:

(10) $$\left\{\begin{array}{l} A=2a\cdot {\mathrm{rand}}_{1}-a\\ C=2{\mathrm{rand}}_{2} \end{array}\right.$$

where *rand*_1_ and *rand*_2_ are random numbers uniformly distributed in the interval [0, 1], and *a* is the convergence factor that decreases linearly from 2 to 0 over the course of iterations.

(11) $$a=2-\frac{2t}{T_{\max}}$$

where *T*_max_ denotes the maximum number of iterations.

2. Spiral bubble-net attacking stage. During this stage, whales simultaneously shrink the encircling circle around the prey and ascend along a spiral path. This behavior is modeled by two mechanisms: (1) shrinking encircling, which drives the swarm toward the optimal solution through the linear decrease of parameter *a*, and (2) spiral position update, which simulates the helical movement of the whales to refine individual positions. The mathematical model is described as follows:

*D*_2_ = |*X_best_*(*t*) − *X*(*t*)| (12)

*X*(*t* + 1) = *D*_2_ ⋅ *e^bl^* ⋅ cos2*πl* + *X_best_*(*t*) (13)

where *D*_2_ represents the distance between the current whale individual and the optimal solution, *b* is the constant determining the shape of the logarithmic spiral, and *l* is a random number uniformly distributed in the interval [−1, 1].

During the hunting process, each whale individual selects either the shrinking encircling mechanism or the spiral position update mechanism based on the value of the random probability *p*, which is mathematically described as follows:

(14) $$X(t+1)=\left\{\begin{array}{ll} X_{\mathrm{best}}(t)-A\cdot D_{1} & p<0.5\\ D_{2}\cdot e^{\mathrm{bl}}\cdot \cos2\mathrm{\pi l}+X_{\mathrm{best}}(t) & p\geq 0.5 \end{array}\right.$$

where *p* is a random number in the range [0, 1]. When *p* < 0.5, the shrinking encircling mechanism is adopted; when *p* ≥ 0.5, the spiral position update is performed.

3. Random search phase. In this phase, the algorithm implements a random walk mechanism. When the coefficient |*A*| > 1, the whale individual no longer updates its position toward the current optimal solution; instead, it moves toward a randomly selected individual in the population to perform a global search. This enhances the algorithm’s global exploration capability and helps prevent premature convergence to local optima. The position update formula in this phase is given as follows:

*D_rand_* = |*C* ⋅ *X_rand_*(*t*) − *X*(*t*)| (15)

*X*(*t* + 1) = *X_rand_*(*t*) − *A* ⋅ *D_rand_* (16)

where *X_rand_*(*t*) denotes the position vector of the randomly selected whale individual.

4. Fitness function. In blasting operations, effective vibration signals exhibit pronounced energy concentration in both the time and frequency domains, whereas noise manifests as a broadband random distribution. After VMD decomposition, the signal is represented by a set of IMFs, each characterized by a distinct center frequency and bandwidth. Envelope entropy quantifies the regularity of a signal envelope. For an ideal IMF, the envelope is smooth and periodic, corresponding to the minimum entropy value; conversely, for components contaminated by noise or mode mixing, the envelope exhibits pronounced fluctuations and high irregularity, leading to elevated entropy values [32,33]. The selection of minimum envelope entropy as the fitness function for VMD parameter optimization is primarily motivated by the following two advantages: (i) Superior noise robustness: In contrast to kurtosis, which is vulnerable to impulsive shock spikes, and correlation coefficients, which rely on reference signals, minimum envelope entropy relies exclusively on the signal’s intrinsic envelope structure. Consequently, it demonstrates enhanced stability against strong transient shocks and background noise prevalent in blasting signals. (ii) No requirement for prior reference information: Due to the highly variable field conditions at blasting sites, acquiring clean vibration signals as references is often infeasible. As an unsupervised metric, envelope entropy is thus more applicable to real-world engineering scenarios. The definition of envelope entropy *E_p_* is as follows:

(17) $$\left\{\begin{array}{l} E_{p}=-\sum_{j=1}^{N}P_{j}\mathrm{lg}P_{j}\\ P_{j}=a(j)/\sum_{j=1}^{N}a(j) \end{array}\right.$$

where *P_j_* represents the probability distribution of each IMF and *a*(*j*) is the envelope entropy obtained by applying the Hilbert transform to the IMF.

### 2.3. Component Selection Principle Based on MPE

Permutation entropy is a probabilistic analysis method proposed by Bandt et al. to measure the complexity of one-dimensional time series [34]. For blasting vibration signals, single-scale permutation entropy is insufficient to fully characterize the non-stationary and time-varying properties of the signal. To address this limitation, MPE is introduced to extract multiscale features from coarse-grained time series via a weighted averaging approach, thereby better reflecting the randomness and dynamic behavior of the signal [35,36]. The principle of the MPE algorithm is as follows:

1. The original time series *X* = {*x_i_*,*i* = 1,2,…,*N*} is subjected to multiscale coarse-graining to generate the coarse-grained sequence $y_{j}^{\left(s\right)}$:

(18) $$y_{j}^{\left(s\right)}=\frac{1}{s}\sum_{i=\left(j-1\right)s+1}^{\mathrm{js}}x_{i}\;j=1,2,\ldots ,\left[N/s\right]$$

where *N* denotes the sequence length, *s* is the scale factor, and [*N*/*s*] represents the integer operation.

2. Conduct phase space reconstruction for the coarse-grained sequence $y_{j}^{\left(s\right)}$ to derive the reconstructed sequence $Y_{l}^{s}$:

(19) $$Y_{l}^{s}=\left\{y_{l}^{\left(s\right)},y_{l+\tau }^{\left(s\right)},\ldots ,y_{l+(m-1)\tau }^{\left(s\right)}\right\}$$

where *τ* represents the delay time, *m* is the embedding dimension, and *l* denotes the *l*-th reconstruction component, with *l* = 1,2,…,*N* − (*m* − 1)*τ*.

3. Sort the elements in the reconstruction component in ascending order, and take the column index of the elements to form a position sequence, that is

*S*(*r*) = (*j*_1_,*j*_2_,…,*j_m_*)  (20)

where *r* = 1,2,…,*R* (*R* ≤ *m*!).

4. As can be seen from the above formula, there are *m*! possible permutation patterns for the position sequence. Calculate the probability *P_r_* of each position sequence appearing in *Y*, and the permutation entropy *H_PK_* of the time series *X* can be expressed as

(21) $$H_{\mathrm{PK}}=-\sum_{r=1}^{R}P_{r}\ln P_{r}$$

5. Normalize the permutation entropy *H_PK_* to obtain the multiscale permutation entropy *H_p_*:

(22) $$H_{p}=\frac{H_{\mathrm{PK}}}{\ln\left(m!\right)}$$

Prior research has established that noise and clean signals differ substantially in permutation entropy. Characterized by greater randomness, noise signals exhibit elevated permutation entropy values, whereas clean signals manifest more structured variations across different scales, yielding comparatively lower entropy. In multiple vibration signal processing applications based on the VMD-MPE method, a value of 0.6 has been widely adopted as an empirical threshold to discriminate between effective and noisy IMFs [37,38]. Furthermore, related studies have investigated the reasonable selection range for the MPE threshold. Based on analyses of both simulated and measured signals, Ma et al. [39] identified an optimal threshold range of 0.55~0.60 and ultimately adopted a value of 0.60. Yang et al. [40] noted that the MPE threshold typically ranges from 0.4 to 0.6; an excessively high threshold may retain noise-dominated components, whereas an unduly low one risks erroneously eliminating valid components. Therefore, the value of 0.6 serves as a commonly adopted empirical benchmark in the VMD-MPE method, rather than a rigid boundary derived from theoretical deduction. Given that measured vibration signals vary considerably in characteristics such as SNR and frequency components under different experimental conditions, the rationale behind this threshold selection will be examined in greater depth in Section 4.2.

### 2.4. Wavelet Threshold Denoising

At the blasting experiment site, measured vibration signals are often contaminated by significant noise arising from electromagnetic interference, geological conditions, and instrumental errors. This contamination severely compromises signal fidelity. Therefore, denoising is essential to accurately extract the time–frequency characteristics and energy propagation patterns of the signals.

The wavelet threshold denoising method leverages multiresolution analysis to effectively extract localized signal features, making it particularly suitable for transient non-stationary signals. The wavelet threshold denoising procedure consists of three main steps: (i) selecting an appropriate wavelet basis and decomposition level, then decomposing the noisy signal via multiscale wavelet transform; (ii) applying thresholding to the resulting detail coefficients; and (iii) reconstructing the denoised signal through inverse wavelet transform using the thresholded coefficients at all levels.

However, the selection of threshold function has a significant impact on the denoising effect. The traditional hard threshold function exhibits discontinuity at the threshold point, which can introduce spurious oscillations in the denoised signal—a phenomenon known as the pseudo-Gibbs effect. Conversely, the soft threshold function performs linear compression on wavelet coefficients greater than the threshold, resulting in a constant deviation between the original and processed coefficients that degrades reconstruction accuracy [8,9,41]. To overcome the limitations of traditional soft and hard threshold functions, this paper adopts a weighted average threshold function, defined in Equation (23), to process the wavelet coefficients. As a linear combination of soft and hard thresholding functions, it yields an effective thresholding behavior by adjusting the parameter μ. This function not only ensures the continuity of the reconstructed signal but also maintains better consistency with the original signal.

(23) $$\omega_{\mathrm{new}}=\left\{\begin{array}{cc} \left(1-\mu )\omega +\mu \cdot \mathrm{sign}(\omega )(\left|\omega \right|-\lambda \right) & \left|\omega \right|\geq \lambda \\ 0 & \left|\omega \right|<\lambda \end{array}\right.$$

(24) $$\mu =e^{-{\left(\left|\omega \right|-\lambda \right)}^{2}}$$

where *ω* denotes the wavelet coefficient after decomposing the noisy signal, *λ* is the selected threshold, *ω_new_* represents the wavelet coefficient processed by the threshold function, *μ* is the weighting coefficient, and *sign*(⋅) is a symbolic function.

### 2.5. Hilbert Spectrum Analysis

HHT is a classical method for non-stationary signal processing. This algorithm first decomposes the signal into multiple IMFs and then performs the Hilbert transform on each component. The Hilbert spectrum describes the instantaneous time–frequency–energy distribution of the non-stationary signal across different scales [12,42,43].

For the original signal *x*(*t*), it can be decomposed into multiple IMFs *c_i_*(*t*) with narrowband characteristics, whose Hilbert transform is defined as follows:

(25) $$H\left[c_{i}(t)\right]=\frac{1}{\pi }\int_{-\infty }^{\infty }\frac{c_{i}(\tau )}{t-\tau }d\tau$$

where *i* is the order of the IMF, *t* denotes the time variable, and *τ* represents the time interval.

The Hilbert transform emphasizes the local property of *c_i_*(*t*). Based on 1/*t*, the analytic signal *z_i_*(*t*) is constructed as

(26) $$z_{i}(t)=c_{i}(t)+\mathrm{jH}\left[c_{i}(t)\right]=a_{i}(t)e^{j\varphi_{i}(t)}$$

where *a_i_*(*t*) is the instantaneous amplitude and *φ_i_*(*t*) denotes the instantaneous phase.

The instantaneous frequency *f_i_*(*t*) can be obtained by differentiating the instantaneous phase function.

(27) $$f_{i}\left(t\right)=\frac{1}{2\pi }\frac{d\varphi_{i}\left(t\right)}{dt}$$

Taking the squared amplitude as the energy measure, the Hilbert energy spectrum of the original signal *x*(*t*) is given as follows:

(28) $$E_{H}\left(f,t\right)=\sum_{i=1}^{n}{\left|a_{i}\left(t\right)\right|}^{2}\cdot \delta \left[f-f_{i}\left(t\right)\right]$$

## 3. Analysis of Simulated Signals

### 3.1. Construction of Simulated Signals

Studies have demonstrated that the single-hole blasting vibration signals effectively capture the vibration propagation characteristics of the rock mass at the experimental sites [44,45]. Moreover, the error between the single-hole blasting vibration signal fitted by Fourier series and the measured signal is relatively small [46]. Consequently, Fourier series can be reliably employed to construct simulated single-hole blasting vibration signals. The functional expression is as follows:

(29) $$f(t)=a_{0}+\sum_{i=1}^{k}\left[a_{i}\cos\left(\mathrm{i\omega t}\right)+b_{i}\sin\left(\mathrm{i\omega t}\right)\right]$$

where *f*(*t*) corresponds to the synthetic single-hole blast vibration signal, *k* refers to the order of the Fourier series, and *a*_0_, *a_i_*, *b_i_* stand for the Fourier parameters.

Based on the above functional form of the simulation signal and the frequency-domain characteristics of actual blasting vibration signals, simulation signals with fundamental frequencies of 40 Hz, 80 Hz, and 120 Hz were generated. To simulate the noise components in the measured signal, Gaussian white noise was added to the clean simulation signals to generate noisy signals with signal-to-noise ratios (SNRs) of 5 dB, 10 dB, and 15 dB, respectively. The mathematical expressions of simulation signals are given as follows:

(30) $$\left\{\begin{array}{l} f_{1}(t)=0.6\cos\left(2\pi \cdot 40\cdot t\right)+0.3\sin\left(2\pi \cdot 40\cdot t\right)\\ f_{2}(t)=0.7\cos\left(2\pi \cdot 80\cdot t\right)+0.4\sin\left(2\pi \cdot 80\cdot t\right)\\ f_{3}(t)=0.5\cos\left(2\pi \cdot 120\cdot t\right)-0.7\sin\left(2\pi \cdot 120\cdot t\right)\\ f(t)=f_{1}(t)+f_{2}(t)+f_{3}(t)+\mathrm{noise} \end{array}\right.$$

### 3.2. Parameter Optimization and Signal Decomposition

**Figure 1:**
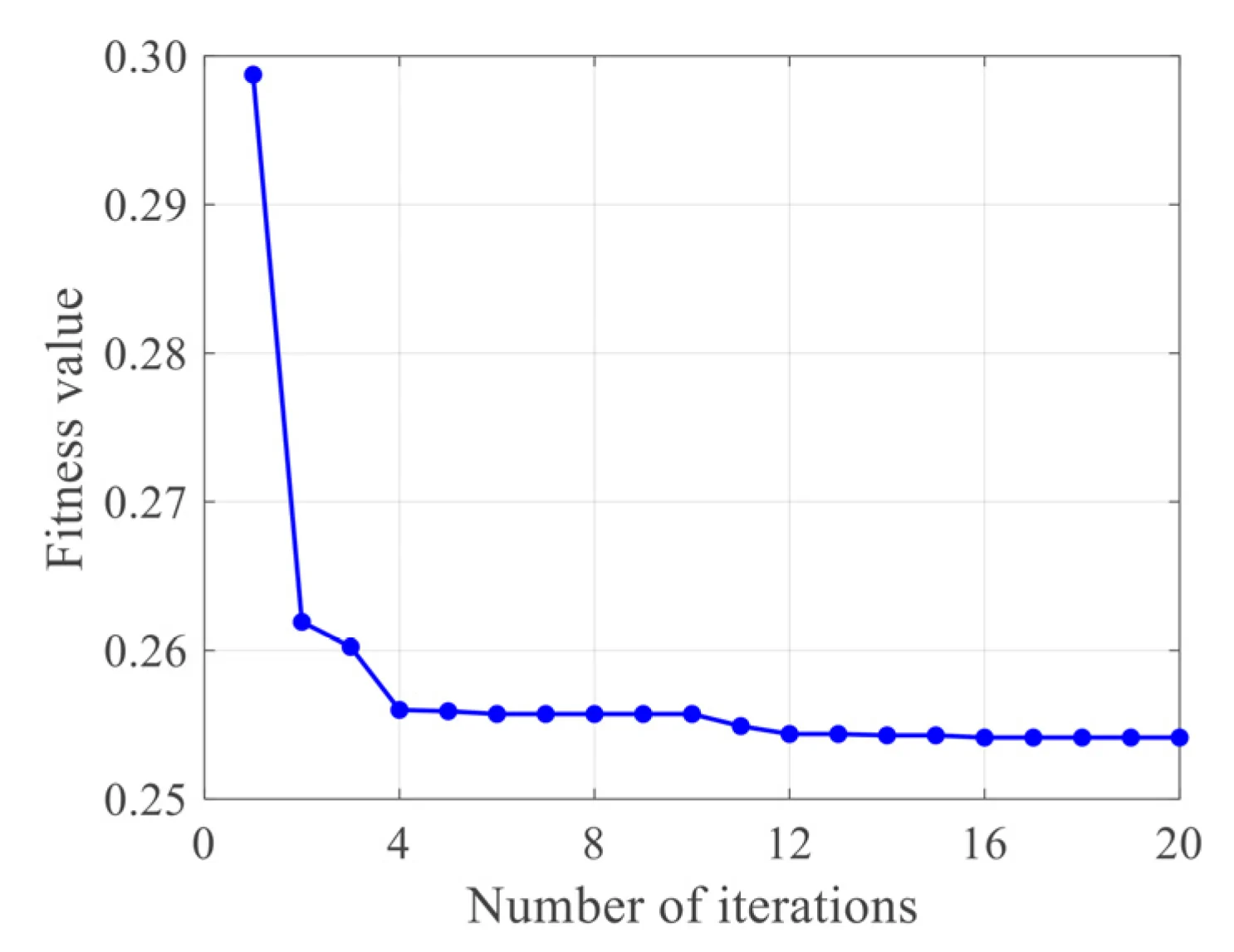
Iterative optimization process of WOA.

Taking the simulation signal with an SNR of 10 dB as an example, the parameter search space for VMD was defined as K ∈ [3, 10] and α ∈ [100, 10,000]. Additionally, the WOA was configured with a population size of 10 and a maximum of 20 iterations, at which point the search process was terminated. During the population initialization phase, a hybrid “chaos + opposition-based learning (OBL)” strategy was employed to avoid the inefficient early-stage search caused by purely random initialization. Specifically, chaotic mapping ensures dense ergodic sampling, while OBL mirrors the search range to double its coverage. The combination of these two techniques maximizes the effective search coverage of the initial population. For the parameter K, a pre-evaluation rounding strategy was adopted: before evaluating the fitness function at each iteration, the value of K is mapped to an integer via the round-off function. The above WOA model was adopted to determine the optimal parameter combination, with the iterative process shown in Figure 1 and the final optimization result yielding K = 8 and α = 5327.

**Figure 2:**
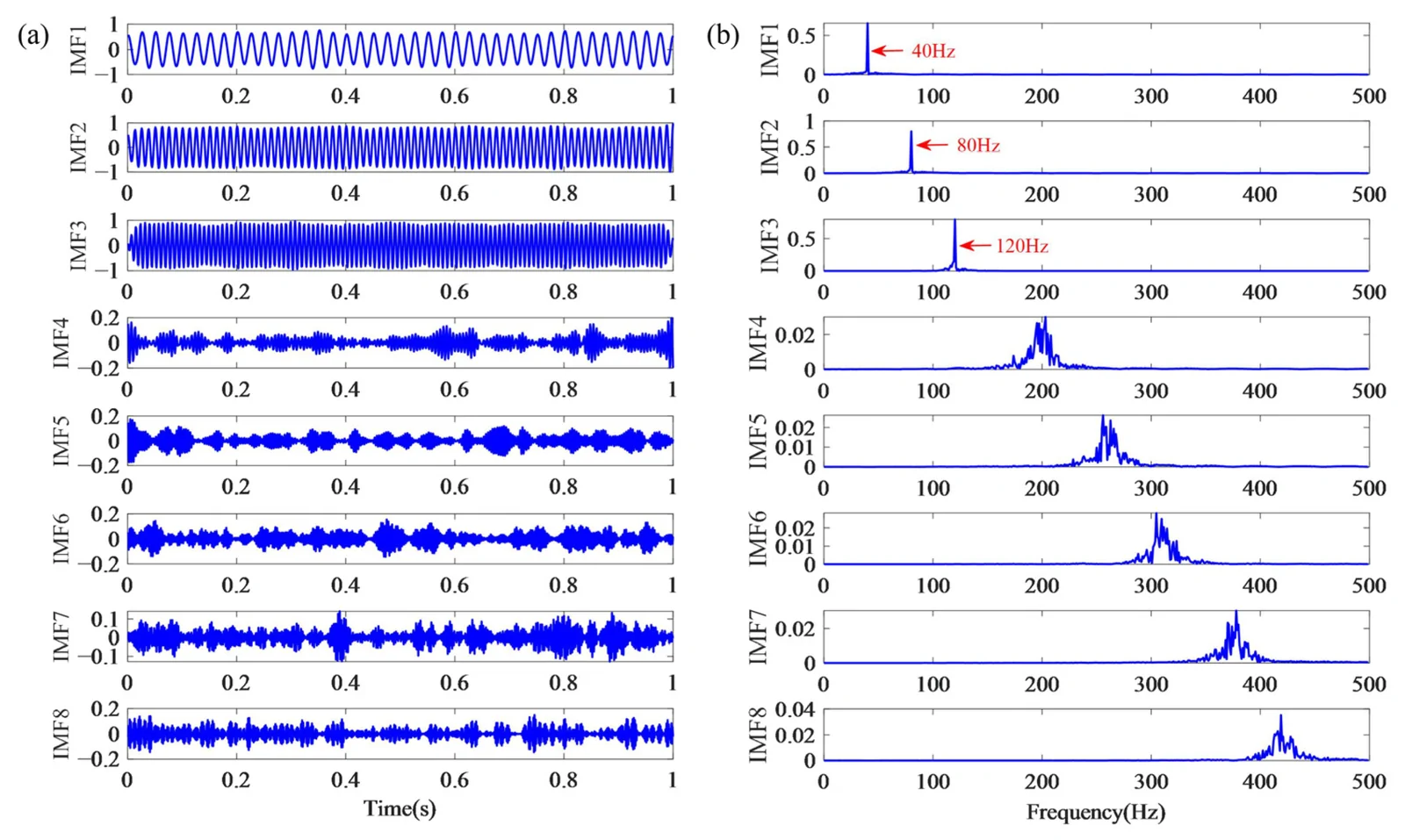
VMD decomposition results of simulated signal. (**a**) IMF time-domain waveforms. (**b**) IMF frequency spectra.

The optimized VMD parameters were applied to decompose the simulated signal, and the resulting IMFs along with their spectra are presented in Figure 2. As can be seen from the figure, the signal was decomposed into eight IMFs. The center frequencies of the first three IMFs are consistent with the fundamental frequencies of the simulated signal, while the remaining components occupy distinct frequency bands, effectively mitigating mode mixing.

### 3.3. Denoising Performance on Simulated Signals

**Figure 3:**
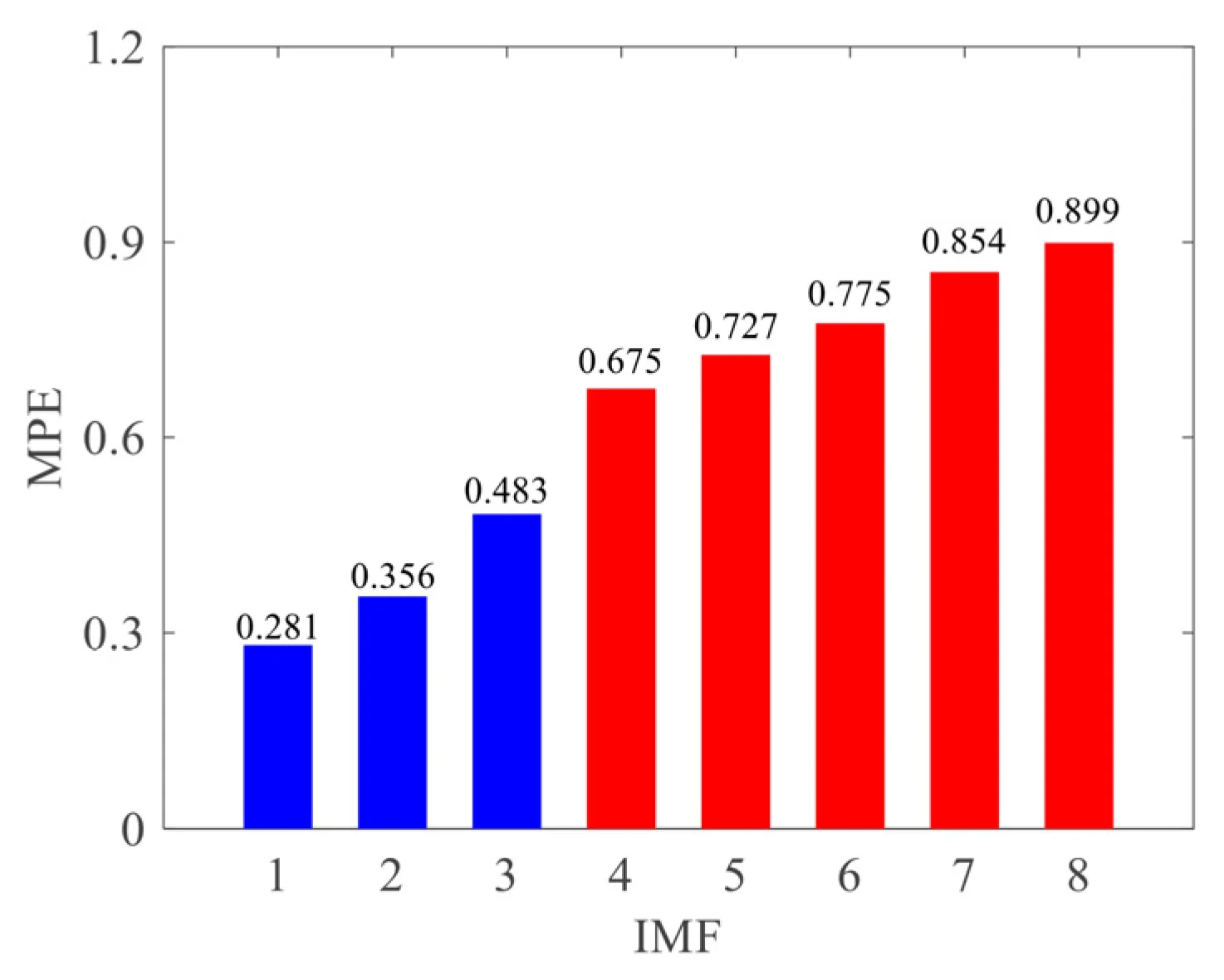
MPE values of IMF components for simulated signal.

According to the MPE-based component selection principle, the MPE of each IMF was calculated, with results presented in Figure 3. From the diagram, it can be seen that MPE values increase monotonically with the IMF order. Specifically, IMFs 1–3 exhibit MPE values below 0.6 and are thus identified as valid signal components, whereas IMFs 4–8 have MPE values exceeding 0.6 and are classified as noise-dominated components.

**Figure 4:**
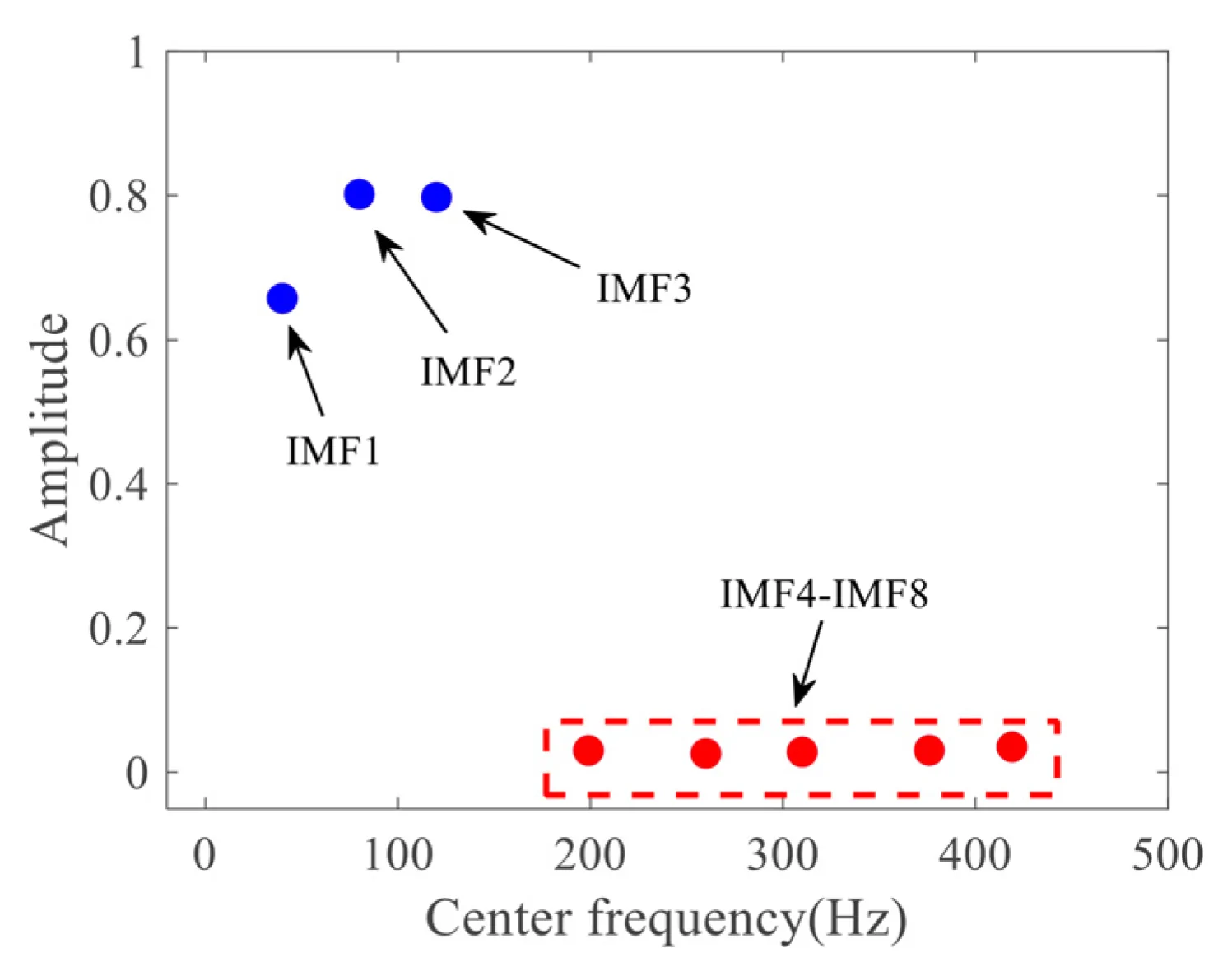
Frequency characteristics of IMF components for simulated signal.

Furthermore, the frequency characteristics of the IMFs were analyzed, with results presented in Figure 4. The last five IMFs, which exhibit higher MPE values and lower amplitudes, correspond to noise components in the simulated signal and are eliminated directly during signal reconstruction. Conversely, the first three IMFs, characterized by lower MPE values and higher amplitudes, align with the three fundamental frequency components of the simulated signal and are retained during signal reconstruction.

**Figure 5:**
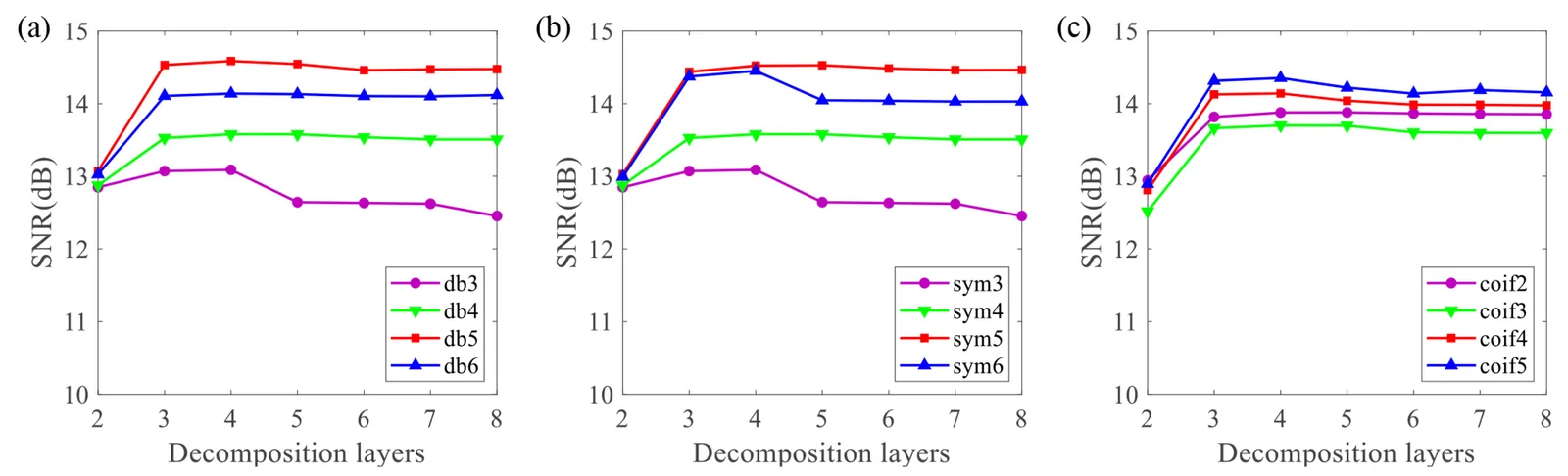
Denoising performance versus decomposition level for (**a**) dbN, (**b**) sym, and (**c**) coif wavelet basis functions.

To further eliminate the noise superimposed on IMFs 1–3, a wavelet threshold denoising model was developed. In this method, the selection of wavelet basis function and decomposition level significantly affects the denoising performance. To identify optimal parameters, three wavelet families, namely dbN, sym, and coif, were selected to decompose the 10 dB simulated signal into 2–8 layers. The resulting SNRs under various combinations are presented in Figure 5. The db5 wavelet basis yields the highest SNR, which peaks at a decomposition level of 4. Accordingly, the configured wavelet threshold denoising model employs the db5 wavelet basis, a decomposition level of 4, a weighted average thresholding function, and the fixed threshold selection rule.

To further evaluate the denoising superiority of the proposed method, a comparative analysis was conducted among four approaches: EMD-wavelet thresholding, EEMD-wavelet thresholding, VMD-wavelet thresholding with preset parameters (K = 6, α = 5000), and VMD-wavelet thresholding with optimized parameters. Three quantitative metrics were employed for performance assessment: SNR, root mean square error (RMSE), and correlation coefficient (COR), defined as follows:

(31) $$S_{\mathrm{NR}}=10\mathrm{lg}\left[\frac{\sum_{i=1}^{n}x_{i}^{2}}{\sum_{i=1}^{n}{\left[x_{i}-y_{i}\right]}^{2}}\right]$$

(32) $$R_{\mathrm{MSE}}=\sqrt{\frac{1}{n}\sum_{i=1}^{n}{\left[x_{i}-y_{i}\right]}^{2}}$$

(33) $$\mathrm{Cor}=\frac{\sum_{i=1}^{n}\left[x_{i}-{\bar{x}}_{i}\right]\left[y_{i}-{\bar{y}}_{i}\right]}{\sqrt{\sum_{i=1}^{n}{\left[x_{i}-{\bar{x}}_{i}\right]}^{2}}\sqrt{\sum_{i=1}^{n}{\left[y_{i}-{\bar{y}}_{i}\right]}^{2}}}$$

where *x_i_* represents the original signal, *y_i_* is the denoised signal, ${\bar{x}}_{i}$ and ${\bar{y}}_{i}$ are the mean values of the two data sequences, and *n* is the number of sampling points.

To eliminate the randomness associated with signal generation and algorithm execution, 20 independent repeated simulations were performed using the above four methods on the simulated signals at 5 dB, 10 dB, and 15 dB SNR. Only the randomly added Gaussian white noise was varied across simulations, with all other parameters held constant. The average values of the three evaluation metrics over the 20 runs are presented in Table 1. As can be seen from the table, for all simulated signals, the optimized VMD-WT method consistently achieved the highest SNR and COR along with the lowest RMSE, demonstrating the best denoising performance. Moreover, the standard deviations of all evaluation metrics for this method across the 20 repeated simulations were significantly smaller than those of the other comparative methods, further confirming that the optimization strategy markedly enhances the stability and repeatability of the algorithm.

Computational burden is measured by the number of decomposition operations; each individual needs one complete VMD run per iteration for fitness evaluation, so a single signal requires at most 200 runs. VMD-wavelet thresholding with preset parameters needs one run but requires *K* and α to be preset empirically, whereas the proposed method determines them adaptively at a higher computational cost. Ensemble methods such as EEMD need no parameter search yet must repeatedly sift numerous white-noise realizations, a comparable effort. The proposed method therefore imposes no greater burden than ensemble decomposition methods, and its parameter search is completed off-line.

### 3.4. Time–Frequency Analysis of Simulated Signals

**Figure 6:**
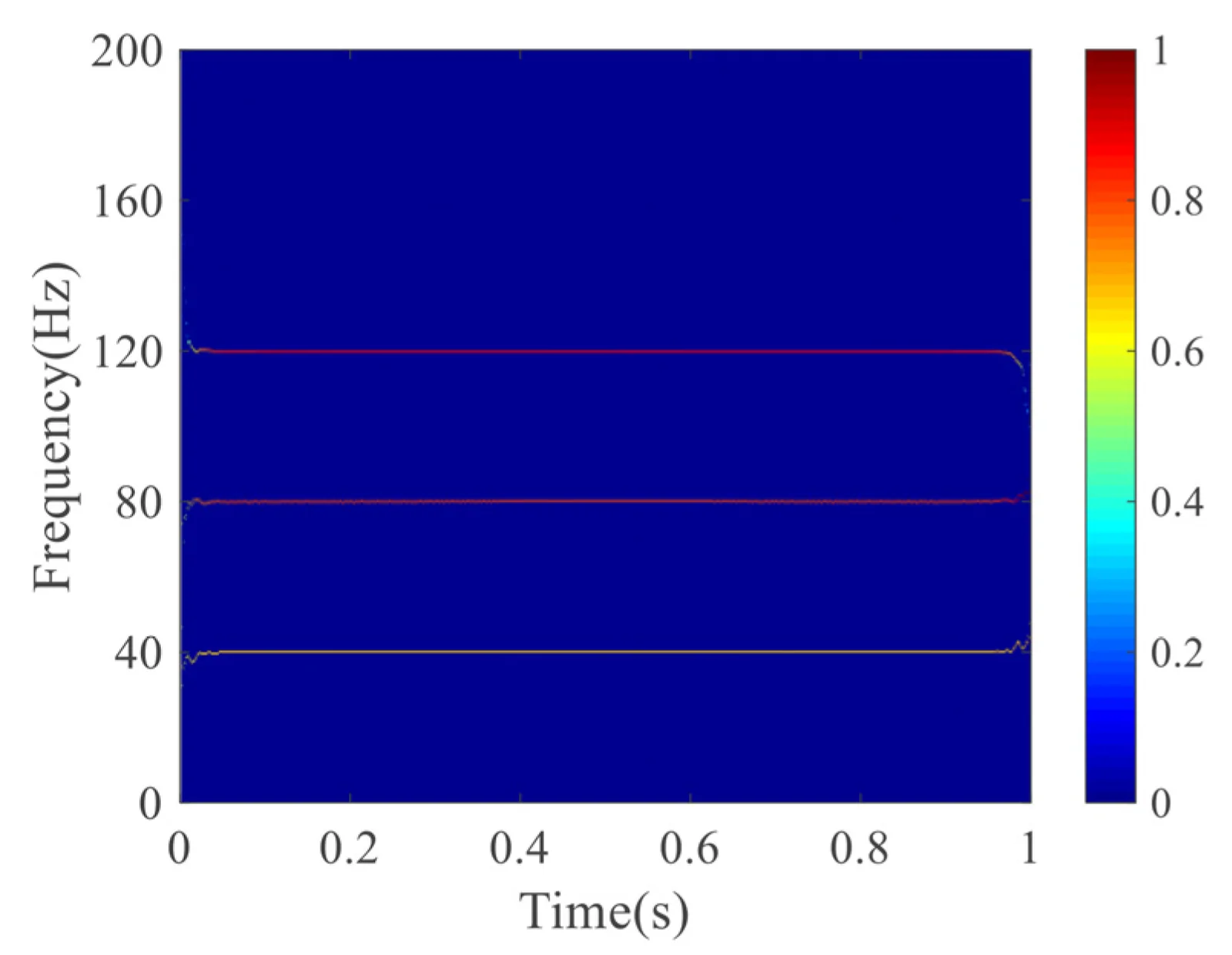
Hilbert time–frequency spectrum of simulated signal.

The signal was reconstructed based on the denoised IMFs 1–3, and the Hilbert spectrum analysis was subsequently performed to obtain the Hilbert time–frequency spectrum (Figure 6). The signal energy is primarily concentrated in frequency bands centered at 40 Hz, 80 Hz, and 120 Hz, accurately reflecting the known spectral characteristics of the simulated signal.

## 4. Analysis of Measured Signal

### 4.1. Vibration Signal Acquisition

**Figure 7:**
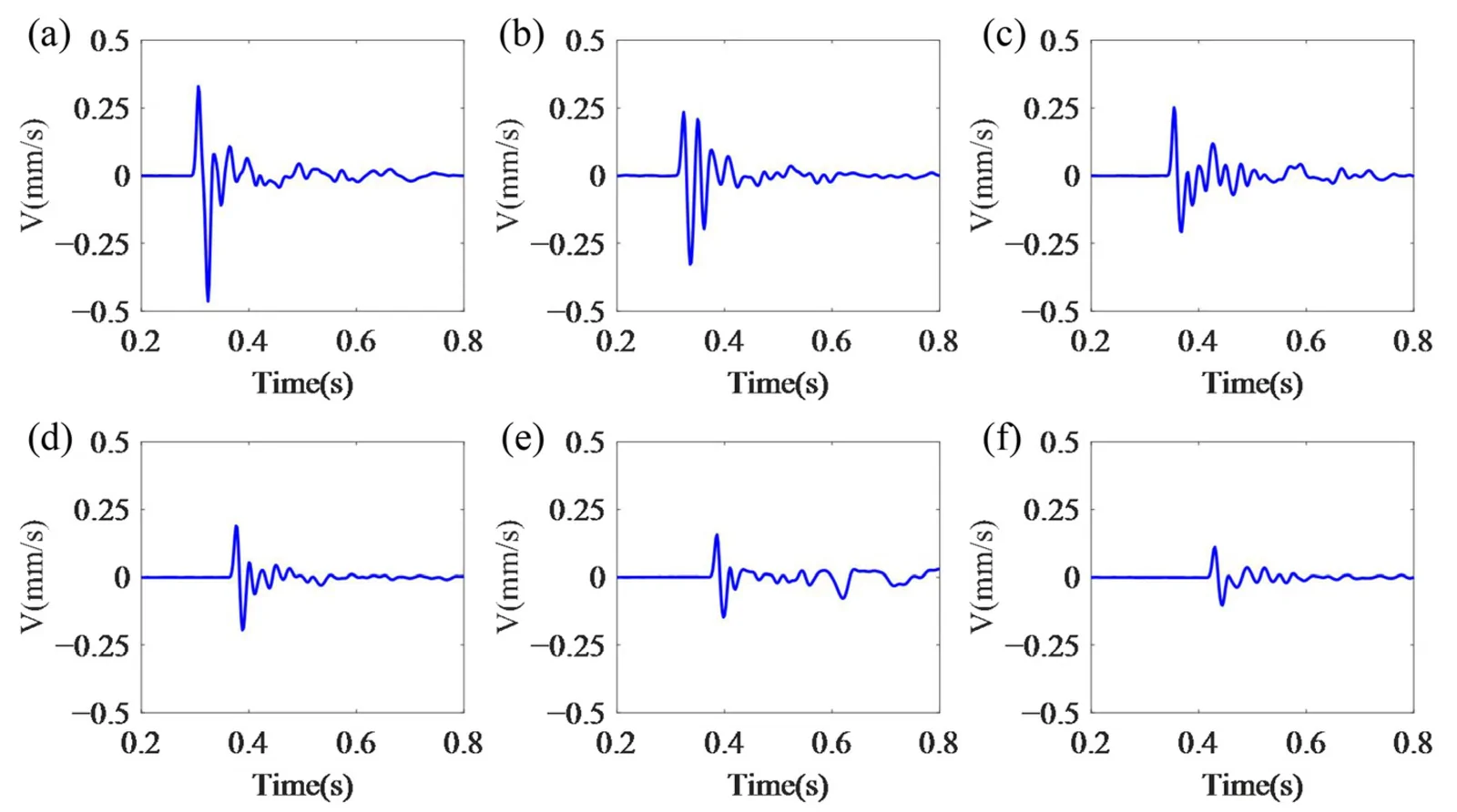
Time-domain waveforms of the measured vibration signals recorded at distances of (**a**) 716 m, (**b**) 747 m, (**c**) 894 m, (**d**) 975 m, (**e**) 983 m, and (**f**) 1188 m from the blast center.

The measured vibration signals were obtained from three single-hole blasting experiments conducted in a coal mine. Vibration monitoring was performed using a velocity-type nodal seismograph with a frequency response range of 0.1–500 Hz and a sampling rate of 1 kHz. The sensors were deployed at distances ranging from 700 to 1200 m from the blasting center. According to the historical experimental data, the seismometer’s frequency response and sampling rate are sufficient to fully capture the effective frequency components of the blasting vibration signals. Using the signal from the third experiment as a representative case, Figure 7 presents the time-domain waveforms recorded at various distances from the blast center.

### 4.2. Signal Decomposition and IMF Selection

The parameter search ranges for VMD were set to K ∈ [6, 16] and α ∈ [100, 10,000], with the WOA algorithm constructed using the same strategy as described in Section 3.2. Subsequently, the VMD model was rebuilt using the optimal parameters, and the measured vibration signals from six monitoring points were decomposed into 10–13 IMFs. The MPE values for each IMF were calculated (Table 2). It should be noted that the decomposition level *K* is determined independently for each measuring point by WOA according to the minimum envelope entropy criterion, rather than being a preset value. As the blast center distance increases, the attenuation of the high-frequency components intensifies and the effective frequency band narrows, so that the number of modes matched to it decreases accordingly.

**Figure 8:**
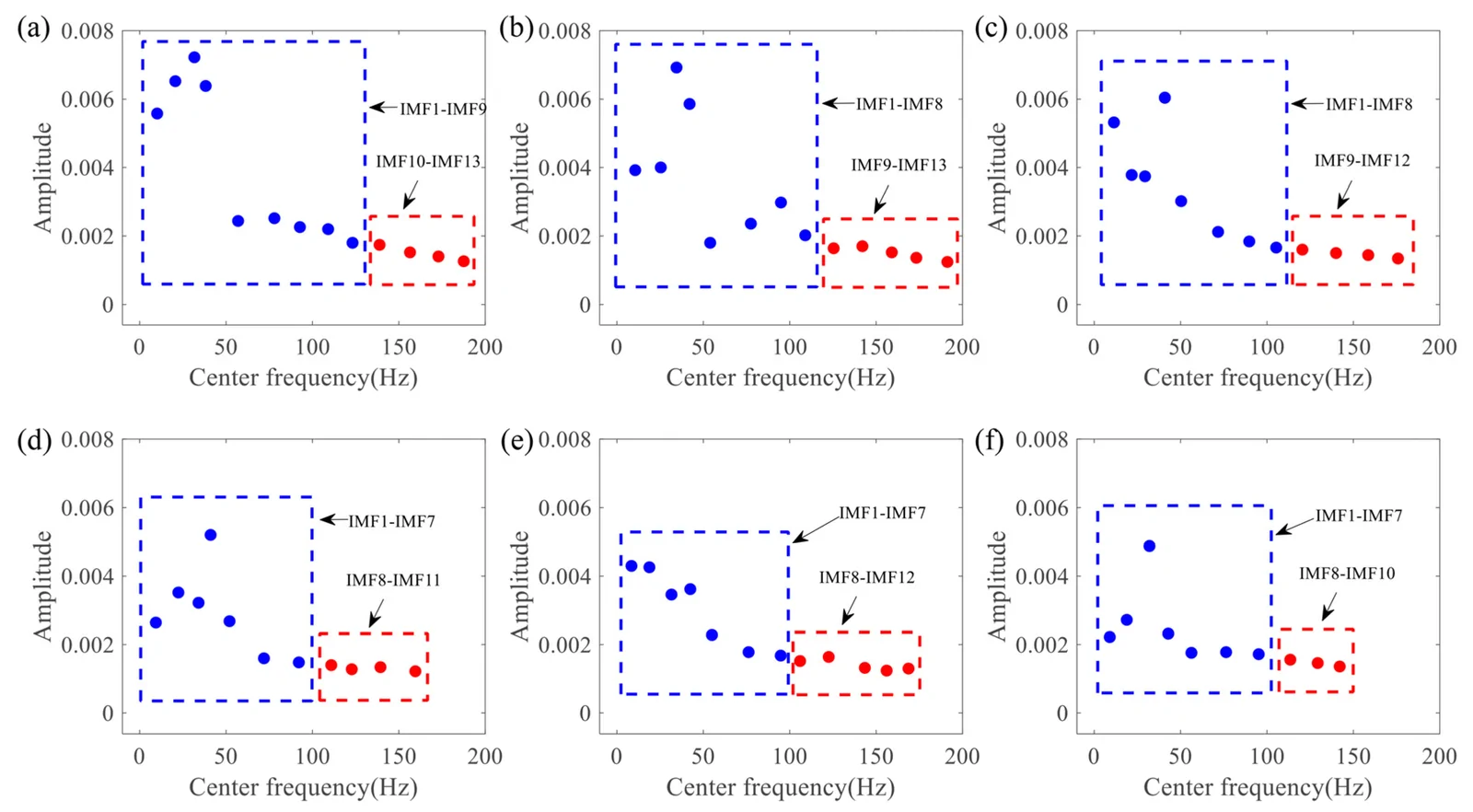
Frequency characteristics of IMFs derived from the measured vibration signals at different distances from the blast center. (**a**) 716 m, (**b**) 747 m, (**c**) 894 m, (**d**) 975 m, (**e**) 983 m, and (**f**) 1188 m.

To verify the influence of the MPE threshold on IMF selection and denoising performance, the values of 0.55, 0.60, and 0.65 were adopted as threshold benchmarks, with the signal energy ratio (SER) and correlation coefficient (COR) before and after denoising serving as evaluation metrics. IMF selection, signal reconstruction, and performance evaluation were conducted on the vibration signals from six monitoring points, with the results presented in Table 3. The analysis indicates that varying the MPE threshold did not cause significant fluctuations in the evaluation metrics of the vibration signals across different blast distances. Taking the signal at 716 m as an example, the threshold variation had no impact on the IMF selection. At the remaining monitoring points, the selection of only one or two IMFs was affected by the threshold adjustment. As can be seen from the frequency characteristics of the IMFs shown in Figure 8, the affected IMFs all exhibit high-frequency and low-energy characteristics, making a negligible contribution to signal reconstruction. In conclusion, varying the MPE threshold within the [0.55, 0.65] range does not significantly affect IMF selection or signal reconstruction, indicating that 0.6 represents a reasonable and robust threshold choice.

### 4.3. Denoising Performance on Measured Signals

**Figure 9:**
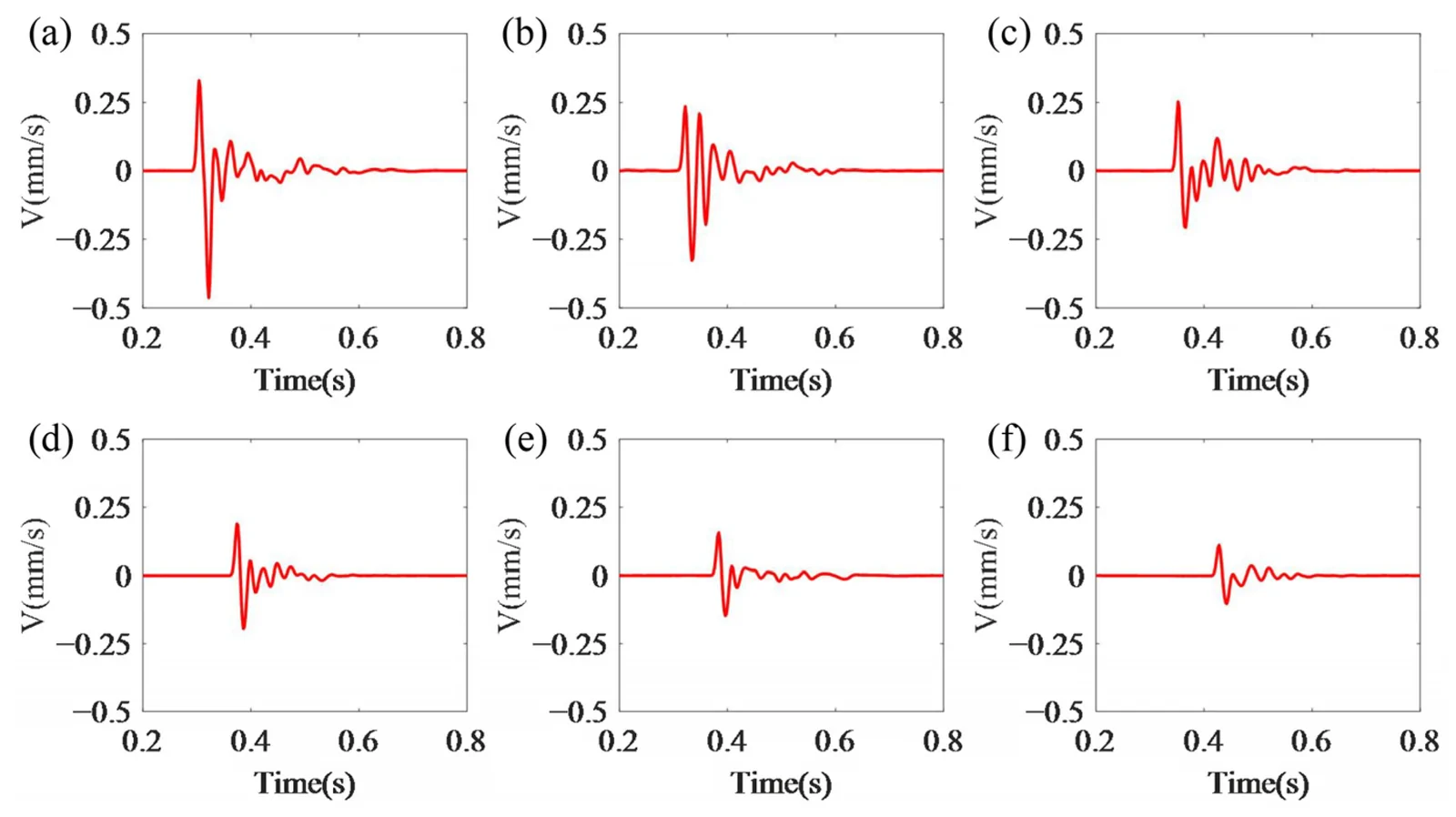
Time-domain waveforms of the denoised signals at distances of (**a**) 716 m, (**b**) 747 m, (**c**) 894 m, (**d**) 975 m, (**e**) 983 m, and (**f**) 1188 m from the blast center.

According to the MPE-based component screening criterion, IMFs with MPE > 0.6 were discarded. The remaining IMFs underwent wavelet threshold denoising followed by signal reconstruction. The time-domain waveforms of the denoised signals are shown in Figure 9. The wavelet threshold parameters adopted for the measured signals are the same as those in Section 3.3 (a db5 wavelet basis, a decomposition level of 4, the weighted average threshold function and the fixed threshold criterion), with no re-tuning. They were determined from the simulated signals rather than selected backwards from the evaluation results of the measured signals. All four methods share identical wavelet parameters, ensuring a fair comparison.

Compared with the original signals in Figure 7, the reconstructed signal effectively suppresses both high-frequency noise and low-frequency trend terms, thereby enhancing waveform clarity and eliminating baseline drift. Regarding signal characteristics, the onset and peak times remain virtually unchanged after denoising. Additionally, the peak velocity exhibits a minor deviation, showing a relative amplitude error of less than 2.5%. This indicates that the reconstructed signal retains the essential features of the original signal with high fidelity.

**Figure 10:**
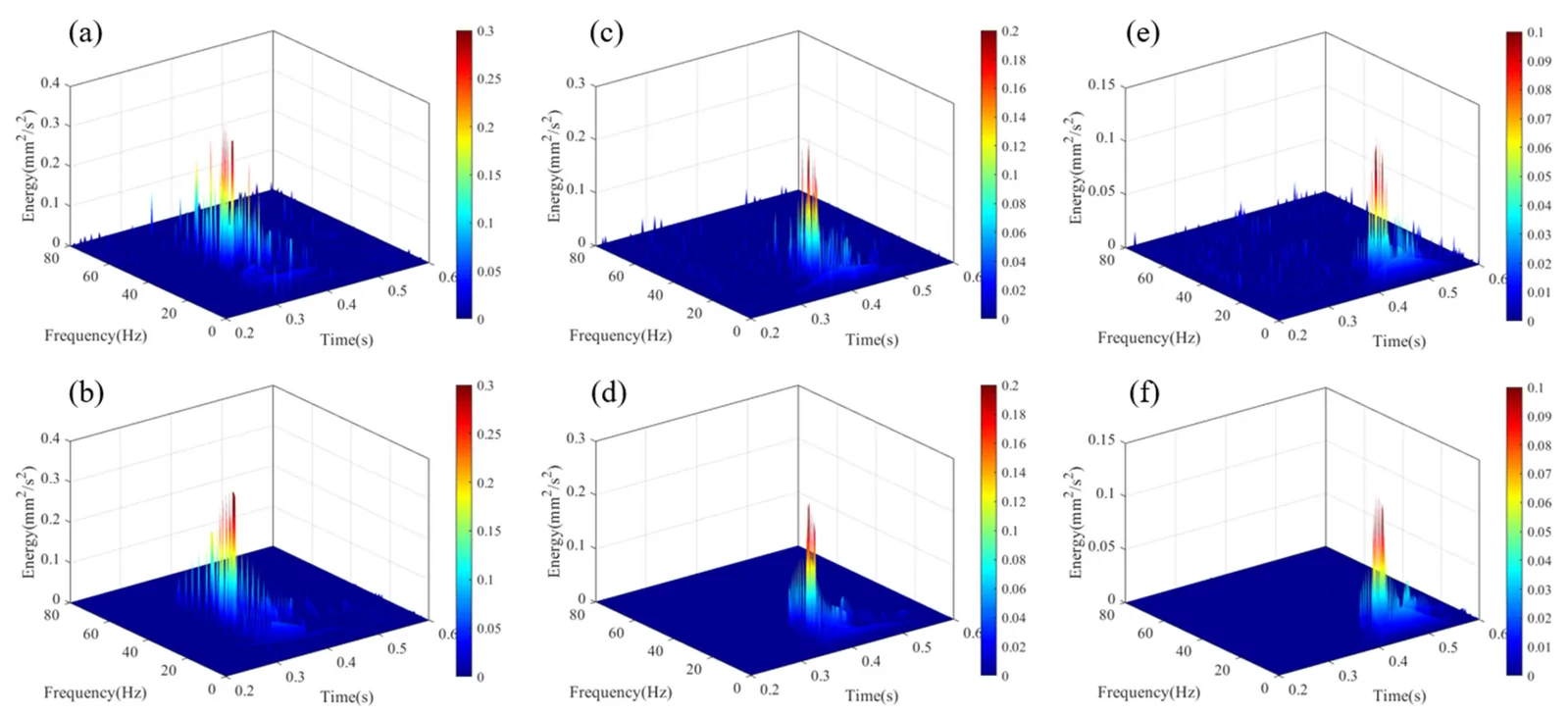
The 3D Hilbert spectrum of vibration signals. (**a**) Raw at 747 m, (**b**) denoised at 747 m, (**c**) raw at 975 m, (**d**) denoised at 975 m, (**e**) raw at 1188 m, and (**f**) denoised at 1188 m.

To further validate the denoising performance, signal characteristics before and after processing were compared in both frequency and energy domains. Taking the vibration signals from three monitoring points (747 m, 975 m, and 1188 m) as representative examples, Figure 10 presents the 3D Hilbert spectra of the original and denoised signals. The denoised signals basically retain the overall trend of the time–frequency characteristics and energy distribution of the originals. Furthermore, key features such as energy concentration period, frequency band, and energy peak remain largely unchanged. The high-frequency noise and low-frequency trend components outside the main frequency interval are effectively suppressed, resulting in a reconstructed signal whose spectral energy is more concentrated within the principal frequency range.

Since the noise-free reference values for measured vibration signals are unavailable, the conventional metrics such as SNR, RMSE, and COR cannot be reliably applied to quantify denoising performance. Therefore, an unsupervised evaluation model was developed with three core indicators: signal energy ratio (SER), signal-to-distortion ratio (SDR), and smoothness (S) [47,48,49].

*SER* evaluates the signal fidelity during the denoising process by calculating the energy difference between the denoised and original signals. A higher SER indicates reduced energy loss, reflecting superior preservation of the underlying vibration characteristics. *SDR* quantifies the noise suppression capability by the ratio of signal deviation to residual noise. The lower SDR signifies smaller deviation from the original signal, corresponding to superior noise suppression performance. *S* reflects the temporal regularity of the denoised signal waveform, and a smaller S indicates weaker local fluctuation, corresponding to a more significant denoising effect. The definitions of these three metrics are given as follows:

(34) $$\mathrm{SER}=\frac{\sqrt{\sum_{i=1}^{n}{y_{i}}^{2}}}{\sqrt{\sum_{i=1}^{n}{x_{i}}^{2}}}$$

(35) $$\mathrm{SDR}=10\mathrm{lg}\left(\frac{\sum_{i=1}^{n}x_{i}^{2}}{\sum_{i=1}^{n}{\left[x_{i}-y_{i}\right]}^{2}}\right)$$

(36) $$S=\frac{\sum_{i=1}^{n-1}{\left[y_{i+1}-y_{i}\right]}^{2}}{\sum_{i=1}^{n-1}{\left[x_{i+1}-x_{i}\right]}^{2}}$$

where *x_i_* denotes the original signal, *y_i_* is the denoised signal (i = 1, 2, …, *n*), and *n* is the number of sampling points.

Four models were sequentially applied to denoise the measured vibration signals: EMD-wavelet thresholding, EEMD-wavelet thresholding, VMD-wavelet thresholding with preset parameters (K = 10, α = 5000), and VMD-wavelet thresholding method with optimized parameters. The statistical results of each evaluation metric are summarized in Table 4. Comparative analysis demonstrates that the optimized VMD-wavelet thresholding method achieves superior performance across all three metrics. Specifically, it yields the highest SER values at all six monitoring points, indicating effective preservation of the signal’s dominant energy components. Regarding SDR, the optimized method produces consistently lower values than competing approaches, confirming its enhanced noise suppression capability. In terms of waveform smoothness, it also attains the optimal result, with the lowest S values recorded at all six points, signifying substantial improvement in temporal regularity and significant reduction of high-frequency noise interference.

In summary, through the comparison of time-domain waveforms, Hilbert time–frequency spectra, and evaluation metrics before and after denoising, it can be concluded that the optimized VMD-wavelet thresholding method exhibits superior adaptability in frequency-domain segmentation and noise component identification. Furthermore, it achieves significant advantages in energy preservation, noise suppression, and waveform smoothness.

### 4.4. Signal Feature Extraction

**Figure 11:**
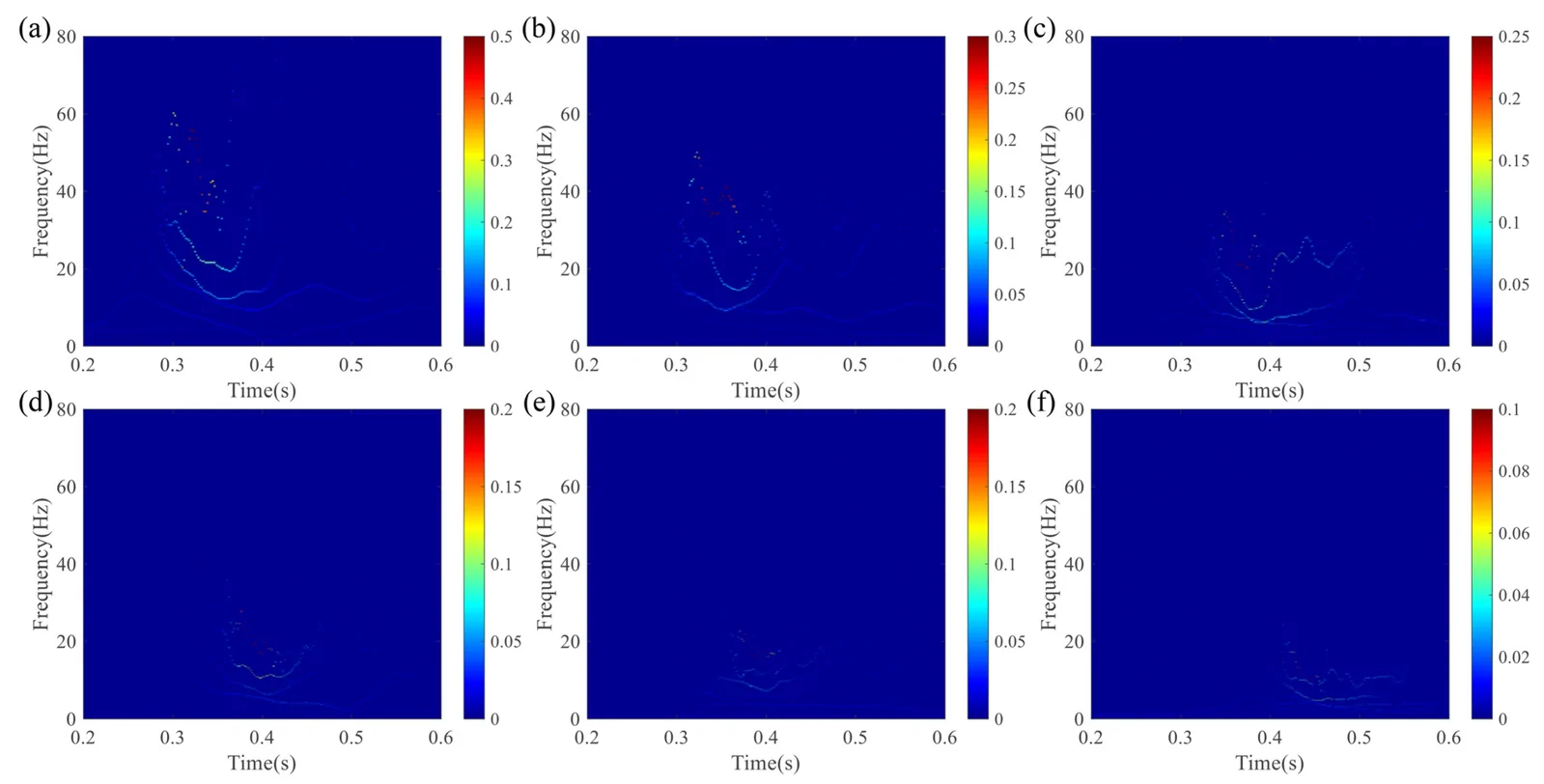
The 2D Hilbert spectrum of the reconstructed signals at (**a**) 716 m, (**b**) 747 m, (**c**) 894 m, (**d**) 975 m, (**e**) 983 m, and (**f**) 1188 m from the blast center.

To visually illustrate how time-domain waveforms, spectral characteristics, and energy distribution evolve with distance from the blasting center, Hilbert spectrum analysis was performed on the reconstructed signals. The 2D Hilbert time–frequency spectra are presented in Figure 11. Based on both the time-domain features and the Hilbert analysis results, Table 5 summarizes the key characteristic parameters of the reconstructed signals at each measuring point, including onset time, peak velocity, peak arrival time, energy distribution interval, and frequency band. In the time domain, signal energy is primarily concentrated within the interval of 0.3–0.5 s. As distance from the blasting center increases, the onset time, peak arrival time, and energy concentration interval are progressively delayed, while both signal amplitude and instantaneous energy decay rapidly. In the frequency domain, the signal energy is predominantly concentrated in the frequency band of 0–60 Hz. With increasing distance, the high-frequency components in the signal attenuate gradually. At distal monitoring points, the attenuation rate of the dominant frequency band diminishes, with energy predominantly concentrated below 20 Hz.

## 5. Discussion

**Figure 12:**
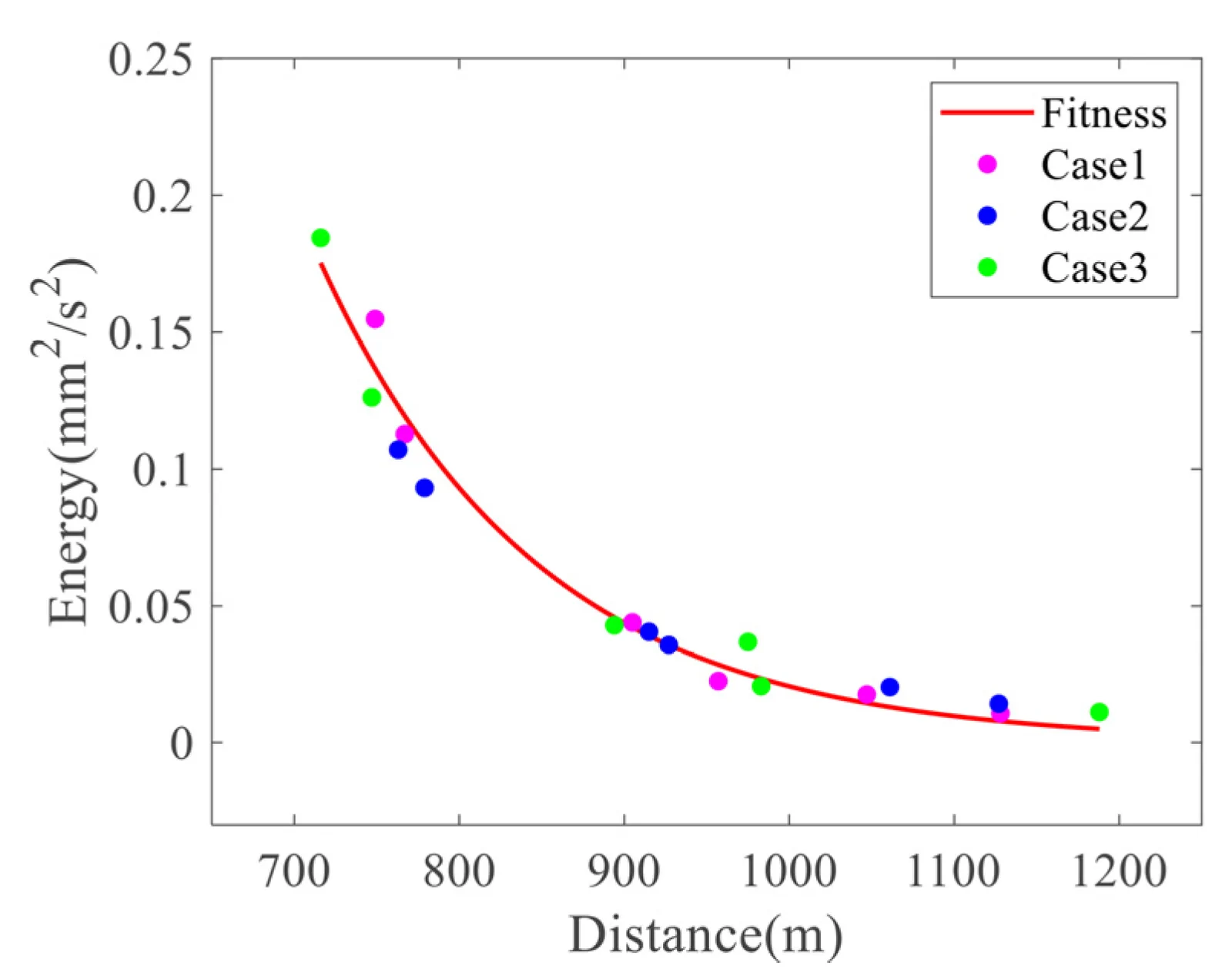
The variation trend of energy peak with blast distance.

A comprehensive analysis was conducted on how energy peak, peak time, and center frequency vary with blast distance across all monitoring points in three experiments. As illustrated in Figure 12, the energy peak exhibits an approximate exponential decay as the distance increases, with consistent trends observed across all three experiments. Within the range of 700–900 m, the energy peak decreases most steeply, and the absolute attenuation per unit distance diminishes significantly beyond this zone. Least-squares fitting of the 18 measured values (three experiments at six blast distances) over the range of 700–1200 m gives

*E* = 39.49 ⋅ *e*^−0.0076⋅^*^D^* (37)

where *D* is the blast distance and *E* is the energy peak. The coefficient of determination is *R*^2^ = 0.9713 and the RMSE is 0.0095.

**Figure 13:**
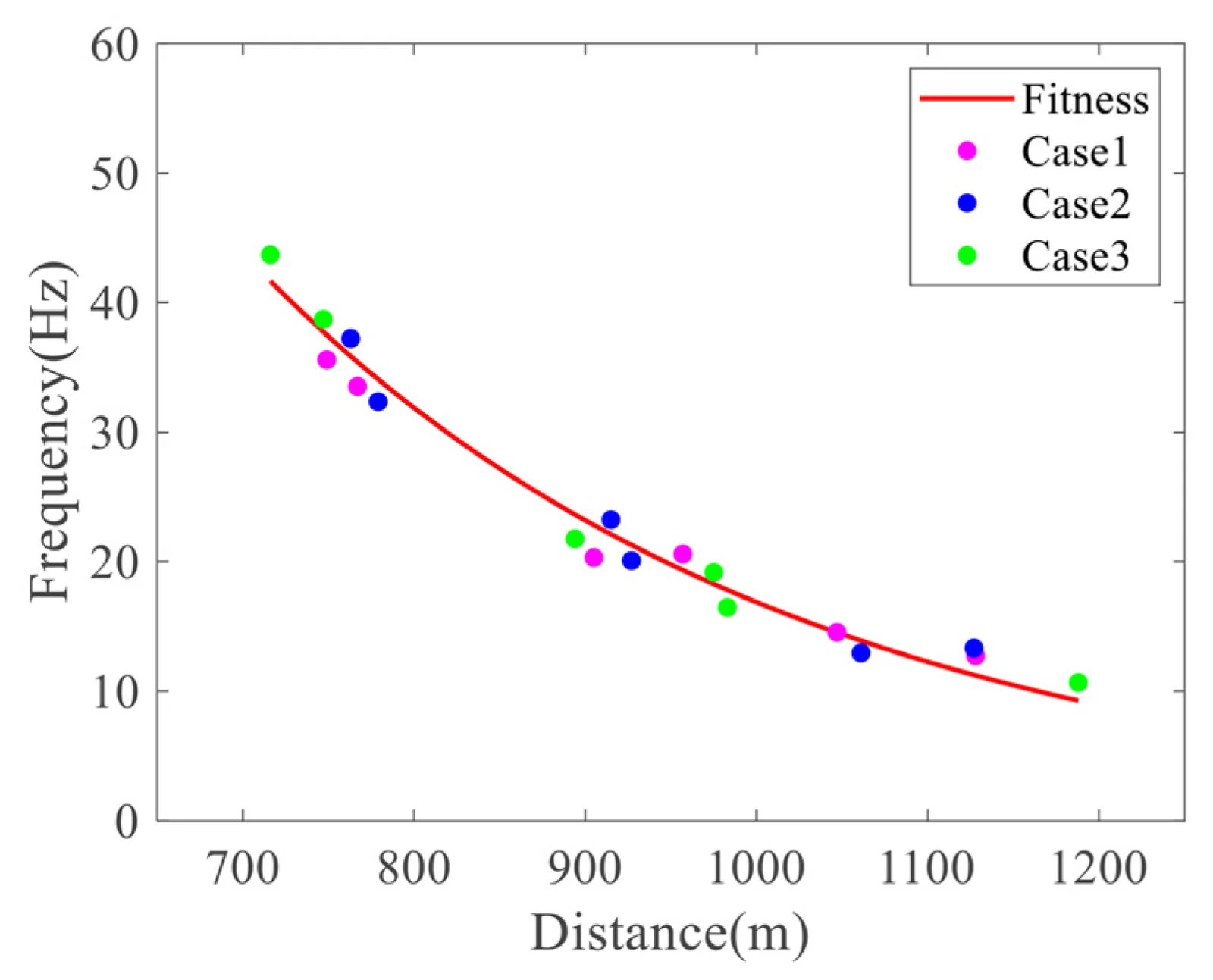
The variation trend of the center frequency with blast distance.

The variation of the signal center frequency with blast distance was analyzed, as shown in Figure 13. The center frequency also exhibits an approximately exponential variation rule with increasing blast distance; however, the overall trend remains relatively stable, and the variation rate is significantly slower than the attenuation process of the energy peak. Applying the same least-squares fitting yields

*F* = 407.49 ⋅ *e*^−0.0032⋅^*^D^* (38)

where *F* is the center frequency and *D* is the blast distance as defined above. The coefficient of determination is *R*^2^ = 0.9901 and the RMSE is 1.07.

**Figure 14:**
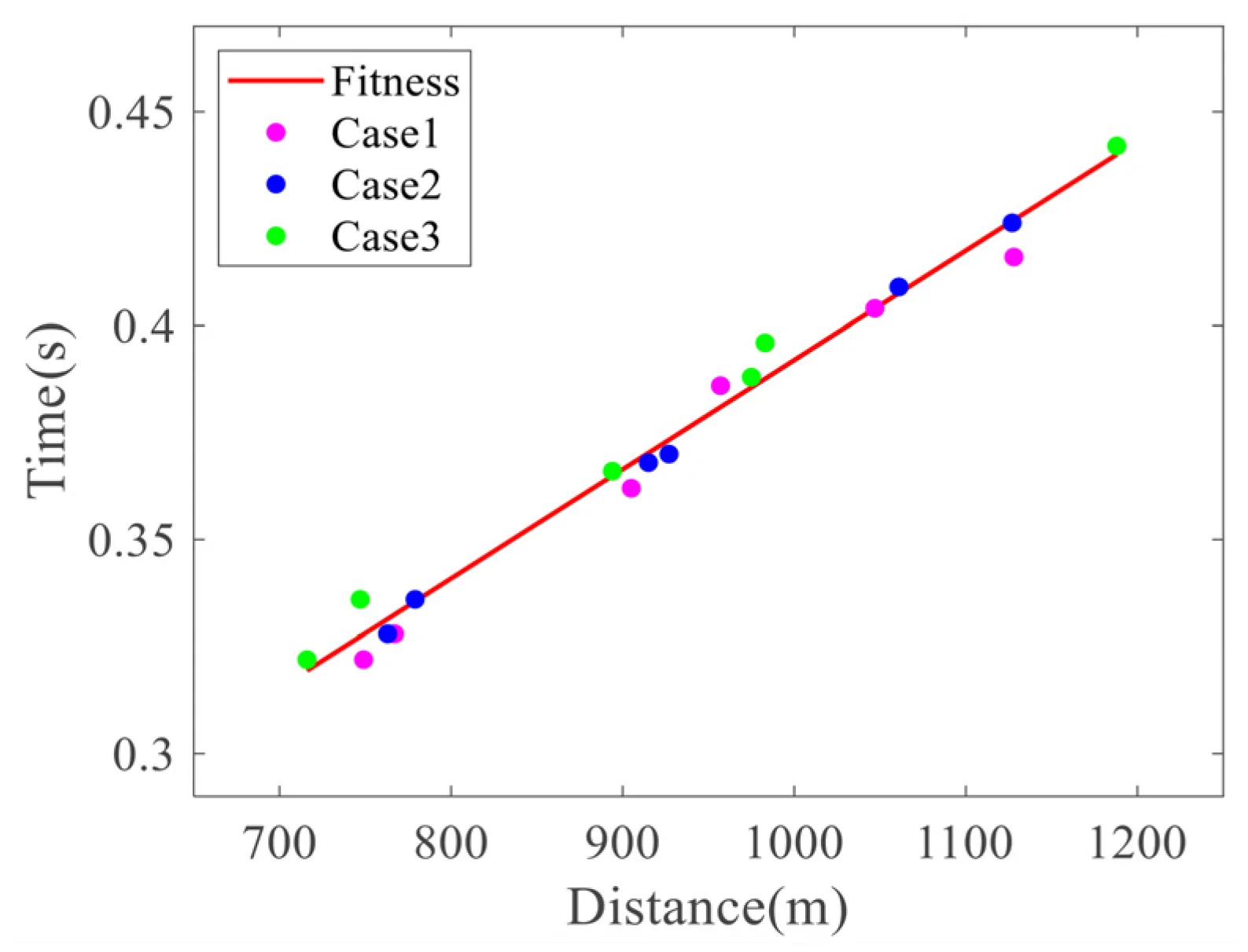
The variation trend of peak time with blast distance.

As illustrated in Figure 14, the peak time exhibits an approximately linear relationship with increasing distance from the blasting center, and the trend is consistent across all three experiments. The wave velocities estimated from this linear relationship show high agreement with the measured values at the experimental site. For the peak time, the corresponding fit is

*T* = 2.56 ⋅ 10^−4^ ⋅ *D* + 0.1365 (39)

where *T* is the peak time and *D* is the blast distance. The coefficient of determination is *R*^2^ = 0.9831 and RMSE = 0.0051.

The physical mechanism governing the above-mentioned patterns can be systematically elucidated through the theory of stress wave propagation in viscoelastic media. The energy attenuation of blast-induced vibration is governed jointly by geometric attenuation and medium absorption. Geometric attenuation is a non-dissipative energy redistribution process: assuming total energy conservation, as the wavefront area expands with propagation distance, the energy flux density per unit area decays according to a power law. Conversely, medium absorption is an irreversible energy dissipation process. It primarily involves three mechanisms: internal friction within the rock mass, frictional sliding along microcrack surfaces, and pore-fluid dissipation. These mechanisms irreversibly transform elastic energy into heat, causing both amplitude and energy to decay exponentially. 

In the blasting experiments presented in this study, the gradient of geometric attenuation remains relatively gentle within the range of 700–1200 m. Meanwhile, the exponential decay law of the energy peak exhibits an excellent fit, indicating that medium absorption is the dominant mechanism of energy attenuation within this distance. Due to the positive frequency dispersion of the medium’s absorption coefficient, high-frequency components are preferentially attenuated during propagation, causing the spectral centroid to continuously shift toward lower frequencies and the center frequency to decrease exponentially with distance. Moreover, since it reflects the evolution of the overall spectrum, its decay rate is more gradual when compared with that of the energy peak. Meanwhile, the peak time increases linearly with distance, indicating that the main energy envelope propagates at an approximately constant velocity. This reflects the kinematic characteristics of the wavefront at the macroscopic scale. 

In summary, the fitting laws of the energy peak, center frequency, and peak time correspond to three independent physical dimensions in the propagation of stress waves: amplitude dissipation, spectral filtering, and wavefront kinematics. The fitting parameters in these models possess explicit physical connotations, rather than being merely empirical statistical constants.

## 6. Conclusions

To investigate the instantaneous distribution characteristics of time, frequency, and energy of blasting vibration signals, as well as their variation patterns with the blast distance, a feature extraction method based on WOA-VMD and Hilbert spectrum analysis was proposed. Taking the single-hole blasting experiments implemented in a coal mine as the case study, this method was applied to the measured vibration signals. The main conclusions are as follows:

1. The WOA algorithm demonstrates excellent iterative convergence speed and robust global search capability during parameter optimization. With optimized parameters, VMD adaptively achieves precise division of signal frequency bands, avoiding the mode mixing or noise redundancy caused by manual empirical parameter selection, and effectively improving the efficiency and accuracy of signal decomposition.
2. The MPE-based component selection criterion enables quantitative identification of noise-dominated components, eliminating random errors introduced by subjective judgment. Furthermore, the wavelet threshold denoising method based on the weighted average threshold function mitigates the defects of soft and hard threshold functions. While ensuring the continuity of the reconstructed signal, it also guarantees consistency between the original and denoised signals. For the simulated signal with an input SNR of 15 dB, the proposed method attains an SNR of 23.31 dB, an RMSE of 0.0786 and a COR of 0.9967, outperforming other comparative methods. For the measured signals from the six monitoring points, the proposed method achieves SER values of 0.931–0.987 and S values of 0.8993–0.9638, ranking first at every point.
3. By analyzing the Hilbert time–frequency spectra of the measured vibration signals, the instantaneous distribution characteristics of signal time, frequency, and energy, as well as their variation patterns with blast distance, were obtained. Within the range of 700–1200 m, the signal energy is predominantly concentrated in the 0.3–0.5 s interval and the 0–60 Hz frequency band. With the increase of the blast distance, both the energy peak and center frequency exhibit exponential decay, while the peak time increases linearly.
4. It is worth objectively acknowledging that the methodology presented herein still leaves considerable scope for enhancement in the following areas. First, the current parameter optimization process requires the repeated execution of VMD, resulting in a significantly higher computational burden compared with other parameter-free methods. In future work, we intend to improve the optimization strategy to reduce the frequency of VMD calls while maintaining optimization accuracy, thereby lowering the overall computational cost. Second, the selection criteria for IMFs require further optimization. Constructing a fusion method based on MPE and the cross-correlation coefficient will be the focus of future work, aiming to provide a more comprehensive theoretical basis for IMF selection. Third, the optimization mechanism of WOA will be further improved. The goal is to enhance the optimization efficiency and reduce ineffective iterations, while maintaining its global search capability.

## Figures and Tables

**Table 1:** Comparative analysis of denoising performance over 20 repeated simulation trials.

Denoising Method		5 dB			10 dB			15 dB	
	SNR	RMSE	COR	SNR	RMSE	COR	SNR	RMSE	COR
EMD-WT	9.4215	0.3642	0.9312	14.8562	0.1975	0.9605	18.7934	0.1182	0.9731
EEMD-WT	10.9834	0.2815	0.9405	16.1125	0.1576	0.9662	20.2547	0.1058	0.9848
VMD-WT (preset)	11.8952	0.2548	0.9585	16.7218	0.1468	0.9701	20.7653	0.0972	0.9867
VMD-WT (optimized)	13.2847	0.1765	0.9828	19.6852	0.1208	0.9918	23.3128	0.0786	0.9967

**Table 2:** The MPE values of IMFs for the measured vibration signals.

Blast Distance	MPE												
	IMF1	IMF2	IMF3	IMF4	IMF5	IMF6	IMF7	IMF8	IMF9	IMF10	IMF11	IMF12	IMF13
716 m	0.1065	0.1577	0.1785	0.2514	0.3427	0.4275	0.4019	0.5148	0.5484	0.6645	0.6909	0.7766	0.7214
747 m	0.0985	0.1793	0.1938	0.2658	0.3323	0.4420	0.5604	0.5925	0.6573	0.6717	0.6998	0.8215	0.8951
894 m	0.0857	0.1681	0.1785	0.2498	0.3026	0.3891	0.4839	0.5756	0.6448	0.6689	0.6945	0.7334	—
975 m	0.0745	0.1741	0.2114	0.2798	0.4744	0.4900	0.5408	0.6485	0.6933	0.7149	0.7665	—	—
983 m	0.1249	0.1886	0.2282	0.3435	0.4167	0.4741	0.5632	0.6492	0.7025	0.7673	0.8239	0.8094	—
1188 m	0.0691	0.1211	0.2582	0.2992	0.3463	0.4654	0.5745	0.6525	0.6305	0.6945	—	—	—

**Table 3:** Comparative evaluation of vibration signal denoising effects under different MPE thresholds.

Blast Distance	MPE = 0.55			MPE = 0.60			MPE = 0.65		
	Valid IMFs	SER	COR	Valid IMFs	SER	COR	Valid IMFs	SER	COR
716 m	IMF 1–9	0.9485	0.9842	IMF 1–9	0.9485	0.9842	IMF 1–9	0.9485	0.9842
747 m	IMF 1–6	0.9421	0.9816	IMF 1–8	0.9475	0.9811	IMF 1–8	0.9475	0.9811
894 m	IMF 1–7	0.9485	0.9854	IMF 1–8	0.9532	0.9875	IMF 1–9	0.9568	0.9882
975 m	IMF 1–7	0.9398	0.9808	IMF 1–7	0.9398	0.9808	IMF 1–8	0.9456	0.9835
983 m	IMF 1–6	0.9324	0.9765	IMF 1–7	0.9385	0.9798	IMF 1–8	0.9421	0.9812
1188 m	IMF 1–6	0.9521	0.9886	IMF 1–7	0.9578	0.9914	IMF 1–7, 9	0.9612	0.9905

**Table 4:** Comparison of denoising performance across different methods.

Blast Distance		SER				SDR				S		
	EMD-WT	EEMD-WT	VMD-WT (Preset)	VMD-WT (Optimized)	EMD-WT	EEMD-WT	VMD-WT (Preset)	VMD-WT (Optimized)	EMD-WT	EEMD-WT	VMD-WT (Preset)	VMD-WT (Optimized)
716 m	0.866	0.951	0.924	0.965	21.697	18.236	19.417	16.385	0.9827	0.9702	0.9765	0.9638
747 m	0.879	0.926	0.947	0.979	19.320	16.280	17.012	15.282	0.9549	0.9271	0.9450	0.9235
894 m	0.821	0.916	0.894	0.942	20.962	16.826	16.344	15.483	0.9643	0.9554	0.9508	0.9492
975 m	0.923	0.965	0.952	0.987	17.551	15.275	14.840	13.481	0.9411	0.9282	0.9326	0.9110
983 m	0.834	0.895	0.887	0.931	18.249	16.883	16.154	14.669	0.9500	0.9426	0.9402	0.9349
1188 m	0.915	0.938	0.960	0.984	15.082	12.855	13.217	12.121	0.9383	0.9160	0.9015	0.8993

**Table 5:** Comparison of time–frequency characteristics and energy distribution parameters across various measuring points.

Blast Distance (m)	Onset Time (s)	Peak Velocity (mm/s)	Peak Time (s)	Center Frequency (Hz)	Energy Concentration Period (s)	Energy Concentration Frequency Band (Hz)
716	0.292	0.4430	0.322	43.69	0.292–0.450	0–62
747	0.311	0.3259	0.336	38.77	0.311–0.432	0–50
894	0.340	0.2074	0.366	22.75	0.340–0.512	0–42
975	0.364	0.1947	0.388	18.16	0.364–0.482	0–33
983	0.370	0.1473	0.396	17.45	0.370–0.480	0–24
1188	0.416	0.1105	0.442	11.66	0.416–0.571	0–20

## Data Availability

The data presented in this study are available on request from the corresponding author.
